# Reliability of diaphragmatic ultrasonography to detect diaphragm dysfunction in critically ill patients

**DOI:** 10.1186/2197-425X-3-S1-A452

**Published:** 2015-10-01

**Authors:** M Dres, B-P Dubé, J Mayaux, J Delemazure, H Prodanovic, T Similowski, A Demoule

**Affiliations:** Respiratory and Critical Care Department, Hopital Pitie Salpetriere, Assistance Publique Hôpitaux de Paris, Paris, France; Sorbonne Universités, UPMC Université Paris 06, INSERM, UMRS 1158 Neurophysiologie Respiratoire Expérimentale et Clinique, Paris, France

## Introduction

The gold standard to diagnose diaphragmatic dysfunction (DD) is the measurement of the intra-thoracic depression is response to a bilateral stimulation of the phrenic nerves (Ptr,stim). This technique is costly, requires expertise and is not widely available at the bedside. On the opposite, ultrasonography is easy to perform but has not been compared.

## Objectives

To validate diaphragm ultrasonography as a tool to detect diaphragm dysfunction in mechanically ventilated patients. Ptr,stim was used as the gold standard.

## Methods

This monocentric prospective study was conducted in a 16-beds medical ICU. DD was assessed by two independent observers at three key time points:

1) during the first 24h of controlled ventilation (CV),

2) as soon as patients could tolerate pressure support ventilation (PSV) and

3) the day of the first spontaneous breathing trial (SBT). Ptr,stim was measured in response to bilateral anterior magnetic stimulation of the phrenic nerves. M-mode ultrasonography was used measure

1) the muscle thickening fraction (TF, (defined as the difference between inspiratory and expiratory muscle thickness divided by the expiratory thickness) of the right hemidiaphragm,

2) the maximal right diaphragmatic excursion was also recorded. A Ptr, stim < -11 cmH_2_O defined DD.

## Results

Seventy-three critically ill patients were investigated (136 measurements: 54 under CV, 32 under PSV and 40 the day of the SBT). DD was present in 79%, 85% and 60% of the patients under CV, PSV and SBT respectively. Taken together, the measurements performed under PSV and the day of the SBT showed a significant correlation between Ptr,stim and TFd (R^2^ = 0.79, p < 0.001). A TF < 29% had a sensitivity and specificity of 85% and 95% to detect DD (area under the receiver operating characteristics curve 0.93).

There were no significant correlation between Ptr,stim and

1. diaphragm thickness
2. the maximal excursion of the diaphragm.

## Conclusions

Diaphragm ultrasonography is a reliable method to identify DD in mechanically ventilated patients with an active inspiration. A diaphragm TF < 29% is a strong predictor of DD.

## Grants

Martin DRES was supported by Assistance Publique Hôpitaux de Paris

